# Cardiac function evaluation for a novel one-step detoxification product of *Aconiti Lateralis Radix Praeparata*

**DOI:** 10.1186/s13020-018-0219-4

**Published:** 2018-12-17

**Authors:** Ya-nan He, Ding-kun Zhang, Jun-zhi Lin, Xue Han, Ya-ming Zhang, Hai-zhu Zhang, Jin Pei, Ming Yang, Jia-bo Wang

**Affiliations:** 1 State Key Laboratory Breeding Base of Systematic Research, Development and Utilization of Chinese Medicine Resources, Development and Utilization of Chinese Medicine Resources, Chengdu University of Traditional Chinese Medicine, Chengdu, People’s Republic of China; 2 Sichuan Good Doctor Panxi Pharmaceutical Co., LTD, Xichang, China; 3 Central Laboratory, Teaching Hospital of Chengdu University of TCM, Chengdu, People’s Republic of China; 4 China Military Institute of Chinese Medicine, 302 Military Hospital, No. 100 Xisihuan, Beijing, 100039 People’s Republic of China; 5 Department of Pharmacy and Chemistry, Dali University, Dali, People’s Republic of China; 6 Jiangxi University of Traditional Chinese Medicine, No. 18 Yunwan Avenue, Nanchang, 330004 People’s Republic of China

**Keywords:** *Aconiti Lateralis Radix Praeparata*, Detoxification approach, Mitochondrial energy metabolism, Cardiac function, Network pharmacology

## Abstract

**Background:**

*Aconiti Lateralis Radix Praeparata* has been used as the first cardiac drug over a 1000 years in Asian countries. Although most detoxification products are confirmed to be safe, the effect is not potent as desired. In previous study, we designed a one-step detoxification product by fresh cutting and continuously dried, which preserved more water-soluble alkaloids while eliminating toxicity. It is thus necessary to find more in vivo evidence to support its industrial development.

**Methods:**

Initially, network pharmacology was applied to analyze the related pathways of candidate components acting on heart failure diseases. Then, two heart failure models that were induced by propafenone hydrochloride and nimodipine (v/v, 1:1) and were given doxorubicin were carried out to test the cardiac activity. Moreover, the effect on mitochondrial energy metabolism was further assessed.

**Results:**

Network pharmacology results indicated that *Aconiti Lateralis Radix Praeparata* treated heart failure through cAMP signaling pathway, calcium signaling pathway, adrenergic signaling in cardiomyocytes and so on. These pathways were highly correlated with myocardial contractility and mitochondrial energy metabolism. Trials on heart failure rats demonstrated that the novel processed-product could produce a stronger positive inotropic action and increase more *Na*^+^–*K*^+^–*ATPase* and *Ca*^*2*+^–*Mg*^*2*+^–*ATPase* than Heishunpian. Pathological results also revealed the novel one could better restore the morphology of cardiomyocytes and reduce vacuolar lesions. It also could inspire more energy with a lower concentration.

**Conclusions:**

This study provides scientific evidence for the clinical application of new products. It is of great benefit to innovate the industrial detoxification process of Aconitum.

**Electronic supplementary material:**

The online version of this article (10.1186/s13020-018-0219-4) contains supplementary material, which is available to authorized users.

## Background

Heart failure (HF) is the ultimate destination of most cardiovascular diseases. Its mortality is gradually increasing with ageing. It has been one of the serious problems affecting global health [1]. In 2004, Van et al. proposed the concept of remodeling myocardial metabolic, and believed that energy metabolism disorder was one of the key mechanisms of HF [2]. At present, classical drugs for treating HF are neuroendocrine system inhibitors, such as angiotensin converting enzyme inhibitors, cardiac glycoside, β-receptor blockers, and aldosterone antagonists [3]. Although these drugs significantly improve the pathological symptoms, the mortality rate is still high. Traditional Chinese medicine plays an important role in complementary and alternative therapies, especially in cardiovascular disease and cancers [4–6].

*Aconiti Lateralis Radix Praeparata* (ALRP) is the daughter root of *Aconitum carmichaelii* Debx. It is regarded as a magic drug for its severe cardiotoxicity and great cardiac effect. How to achieve the dual purpose of toxicity elimination and efficacy preservation is always a bottleneck of industrial development. Traditional detoxification methods are extremely complicated and time-consuming, such as burning, grilling, baking, boiling, soaking, steaming, and so on [7, 8]. What are worse, over 90% alkaloids lost during the process due to unclear understanding of toxic and active ingredients [9]. Official quality standards of Heishunpian (HSP), Baifupian, Danfupian, Paofupian are cases. From the present perspective, aconitine, mesaconitine, and hypaconitine belong to the hypertoxic main components. When these ones are hydrolyzed under heating conditions, the hydrolysates are converted into possible energy metabolism promoters, including benzoylaconitine, benzoylmesaconitine, and benzoylhypaconitine [10]. Other water-soluble alkaloids, such as higenamine and salsolinol [11, 12], are considered to be crucial substances in enhancing myocardial contractility by activating β receptors [13]. Interestingly, both benzoyl alkaloids and water-soluble alkaloids have good water solubility and are easy to flow away.

According to the principle above, we have designed a novel detoxification approach by fresh cutting and continuously dried in 100 °C oven for 10 h [14]. In the previous study, this new method has been confirmed to achieve the same detoxification effect as traditional methods, and the loss of alkaloids dropped from 85.2 to 30% [14]. The crucial innovation has granted by Chinese patents (No. 201510347673.9). However, there is no direct evidence that the increase of alkaloid retention in vitro would lead to enhancing the effect in vivo. It is unknown whether the novel ALRP processed product (NAP) has a better efficacy on cardiac.

In this manuscript, in order to explore the mechanism of cardiac by ALRP, the methods and ideas of network pharmacology were applied. HSP was the most widely used ALRP detoxification product at present. To evaluate the clinical application advantages of NAP, cardiac activity experiment and mitochondrial promotion experiment were carried out. This study is expected to reveal the cardiac mechanism of ALRP and to facilitate the clinical application of NAP. It is also very important for scientific design of detoxification technology and efficient utilization of ALRP resources.

## Methods

### Materials

HSP and ALRP were provided by *Sichuan Jiangyou Zhongba Fuzi Science and Technology Development Co.*, *LTD.* All samples, identified by Professor Xiaohe Xiao, were deposited at the Chengdu University of TCM, Chengdu, China. NAP was prepared by the one-step detoxification approach. In detail, fresh ALRP were cutting into particles with the size of 5 * 5 * 5 mm, and then dried in 100 °C oven for 10 h [14]. The information regarding the experimental design, statistics, and resources used in this study are attached in the minimum standards of reporting checklist (Additional file 1).

### Chemicals

Coomassie (Bradford) Protein Assay Kit was purchased from Nanjing Jiancheng Bioengineering Institute (A045-2, China). Sucrose was purchased from BeijingYilijingxi Co., Ltd (20071227). Tris–HCl was purchased from BIOPCR (ZH136590). Na_2_EDTA (20100512) and PBS (20150712) were purchased from Solarbio. Bovine serum albumin was purchased from Sigma (#SLBL5598V). Hepes was purchased from Hotaibio (H0070). Propafenone Hydrochloride Injection (PHI) was purchased from Guangzhou Baiyun Shan Ming Xing Pharmaceutical Co., Ltd., and the specification was 10 ml: 35 mg. Nimodipine Injection (NI) was purchased from Bayer Schering Pharma, and the specification was 50 ml: 10 mg. Normal saline (NS) was got from Shijiazhuang No. 4 Pharmaceutical, and the specification was 500 ml: 4.5 g. Adriamycin was purchased from Shenzhen Wanle Pharmaceutical Co., Ltd. and the specification was 10 ml: 20 mg. The ultrapure water used in the experiments was prepared using a Milli-Q Ultrapure water purification system (Millipore, Bedford, MA, USA).

### Animals

Male Sprague–Dawley (SD) rats weighing 180–200 g were obtained from the Laboratory Animal Center of the Military Medical Science Academy of the PLA (Permit No. SCXK-(A) 2012-0004). The animals were maintained under controlled conditions of temperature 20 ± 0.5 °C, humidity 55 ± 5%, and with 12 h light and 12 h dark cycles. Before experiments, they were fasting for 24 h with free access to water.

### Network pharmacology analysis

#### Collect predicted targets of ALRP and known targets of heart failure

Benzoylaconitine, benzoylmesaconitine, benzoylhypaconitine, higenamine and salsoline were selected to explore the information of predicted targets. Information was obtained from BATMAN-TCM [15, 16]. Score cutoff > 20 and P < 0.05 were used as screening parameters to find potential targets of five components [15]. Known therapeutic targets for the treatment of heart failure were obtained from two resources. The first one was the Human Phenotype Ontology (HPO) database [17, 18], and the second one was Therapeutic Target Database (TTD) [19, 20].

#### Protein–protein interaction (PPI) data and Network construction

PPI data were imported from String database [21, 22]. Then gave a score for each PPI data. In the analysis, homo species were chosen. To ensure the reliability, the one with a score of over 0.7 was considered acceptable [23]. Based on PPI data results, Cytoscape software (Version 3.5.1) was applied to visualize the interaction network. Network Analyzer, a plug-in for Cytoscape, was also used to calculate the topological properties [24], and construct an interaction network map of “drug target-disease target” with the target over the median of degrees, betweenness, and closeness.

#### Pathway enrichment analysis for candidate targets

DAVID Bioinformatics Resources 6.8 [25, 26] and KOBAS 3.0 were applied for pathway enrichment analysis [27, 28].

### Effect on an acute heart failure model induced by Propafenone hydrochloride and nimodipine injection

#### Extraction of sample

200 g NAP or HSP were extracted 1 time with 10-fold the amount of water and 1 h each time. The extracted solution was cooled, contributing to weight loss during the extraction procedure, and then centrifuged 10 min with a speed of 5000 rpm min^−1^ to yield the sample solution.

#### Determination method

Eighteen male SD rats were divided into three groups consisting of six animals in each. They were control group, NAP group and HSP group, respectively. Rats were anesthetized using 20% urethane solution through intraperitoneal injection. Rats were placed in dorsal recumbency and a longitudinal midline incision was made in the neck. The right common carotid artery was isolated, and an arterial canal processed by heparin was inserted into the left ventricle. It could monitor the change of left ventricular maximum pressure rising rate (+dp/dtmax). A small incision was cut on the vein, and a venous cannula was inserted. The model drug and extraction were injected into the rats via it. All signals were synchronously recorded on the four-channel physiological recorder (RM6240BD, Chengdu instrument factory). Before the experiment, it was required to correct the pressure transducer using the sphygmomanometer. When measuring the left ventricular pressure, it should turn the three-way valve and close the duct to link the transducer and the atmospheric, after a fast zero correction, we can record data. Propafenone hydrochloride and nimodipine injection (v/v, 1:1) were injected into rats at a constant speed of 4 ml h^−1^. When the +dp/dtmax dropped more than 50%, the injection was stopped. If the value of +dp/dtmax did not rise in 5 min, the model was considered successful. At this time, the extraction of NAP or HSP was given at a constant speed of 10 ml h^−1^. And the rise of +dp/dtmax within 15 min were figured out to evaluate the cardiac effect.

### Effect on a heart failure model induced by Adriamycin

#### Extraction of sample

200 g NAP or HSP were extracted 2 times with 10-fold the amount of water, 1 h each time. The extraction was filtered through a qualitative filter paper and then the filtrate was concentrated to 1 g/ml at 60 °C.

#### Determination method

Twenty-four male SD rats were divided into four groups consisting of six animals in each. They were normal group, model group, NAP group and HSP group, respectively.

Each group was administered continuously for 5 days. Dose volume of 10 ml kg^−1^ extracts was given orally one time each day, while normal group and model group were given the same amount of water. On the sixth day, in addition to the normal group, the other four groups received 10 mg kg^−1^ Adriamycin through single intraperitoneal injection to copy heart failure model [29].

24 h after modeling, rats were anesthetized using 20% urethane solution through intraperitoneal injection. Rats were placed in dorsal recumbency and a longitudinal midline incision was made in the neck. The right common carotid artery was isolated, and an arterial canal processed by heparin was inserted into the left ventricle. After stable 20 min, a polygraph (RM6240BD, Chengdu instrument factory) was used to record left ventricular systolic pressure (LVSP), +dp/dtmax, and heart rate (HR). After the determination of cardiac function, hearts were removed and washed with cold saline water immediately. The contents of* Na*^*+*^–*K*^*+*^–*ATPase* and* Ca*^*2+*^–*Mg*^*2+*^–*ATPase* were determined after homogenization. The protein was quantitatively used in coomassie brilliant blue. This part was commissioned by Google biotechnology limited company.

The left ventricular myocardium was obtained by fixation with 10% formalin fixation fluid. Then it was prepared into a conventional tissue section and the pathological observation was performed after staining HE. This part was commissioned by Pathology Department of 302 Military Hospital.

All values were expressed as mean ± SD. The results were analyzed by one-way analysis of variance (ANOVA) using SPSS 22.0 software. A value of *p *< 0.01 was considered statistically significant.

### Effect on the mitochondrial energy metabolism

#### Instrument

Microcalorimetry (TAM AIR 3114 Bioactivity monitor, Sweden) was utilized to measure the power–time curves of metabolic heat release of mitochondria. The baseline fluctuation was less than 20 μW over 24 h. The high-speed refrigerated centrifuge Sigma 3–18 k (Sigma, Germany) and Homogenizer machine T10 Basic (IKA, Germany) were applied in this research. For details of the performance and structure of the instrument, please see the instruction and Ref [30].

#### Test solution preparation

Buffer A was a mixture of 68.5 g sucrose, 3 g Tris–HCl, 0.18 g Na_2_EDTA, and 0.5 g bovine serum albumin and diluted to 500 ml. Buffer B was a solution of 51.3 g sucrose, 1.2 g Tris–HCl, and 0.1 g Hepes and diluted to 500 ml and sterilized under high temperature and pressure. All chemicals were of analytical grade.

#### Sample preparation

10 g NAP or HSP were extracted 2 times with 10-fold the amount of water and 1 h each time. The extraction was filtered through a qualitative filter paper. The filtrate was prepared to a final concentration of 50 μg ml^−1^ with Buffer B as a solvent.

#### Mitochondria isolation

Mitochondria were isolated from the liver of SD rats killed by exsanguination and cut into small pieces and washed with PBS and Buffer A. Then, the liver tissues were homogenized by homogenizer aseptically. The homogenate was centrifuged at 5000 r min^−1^ for 15 min at 4 °C, and the sediment was discarded. The supernatant was centrifuged at 10,000 r min^−1^ for 20 min at 4 °C, then the sediment was kept. Finally, the sediment was re-suspended with Buffer B to form the mitochondria suspension. The isolated mitochondria were stored at 4 °C, and the concentration was quantified by Coomassie (Bradford) Protein Assay Kit [10].

#### Microcalorimetric measurement

The metabolic heat generation of isolated mitochondria and the thermal effects of NAP and HSP were determined using TAM Air Isothermal Microcalorimetry. The penicillin bottle was processed by strong acid, washed with ultrapure water, and sterilized at 37 °C. Ten milliliters of Buffer B was added into one penicillin bottle as the sterile control group (Ch 1). The same volume of mitochondria suspension was added into the rest seven penicillin bottles, including one blank control group (Ch 2) and five administered groups (Ch 3–7) at different concentrations of NAP or HSP extraction. The information of added solutions of each group was shown in Table 1.

**Table 1:** Reagent addition to ampoule of each channel (mL)

Channel no	Mitochondria suspension/mL	Buffer B, solution/mL	Sample, solution/mL
Ch 1	0	10	0
Ch 2	6	4	0
Ch 3	6	3.95	0.05
Ch 4	6	3.9	0.1
Ch 5	6	3.8	0.2
Ch 6	6	3.6	0.4
Ch 7	6	3	1

Then, the bottles were sealed and put into the TAM Air Isothermal Microcalorimetry. The heat flow curves of each channel were recorded until they returned to a steady state. All data were collected by a dedicated software package in a real-time manner [10]. Principal component analysis (PCA) was performed on the quantitative thermokinetic parameters which were obtained by analyzing the power–time curves of rat liver mitochondria growth affected by NAP and HSP using SPSS 22.0 statistics software (SPSS Inc., Chicago, IL, USA).

The Minimum Standards of Reporting Checklist contains details of the experimental design, and statistics, and resources used in this study.

## Results

### Network pharmacology analysis

#### Network construction

**Fig. 1:**
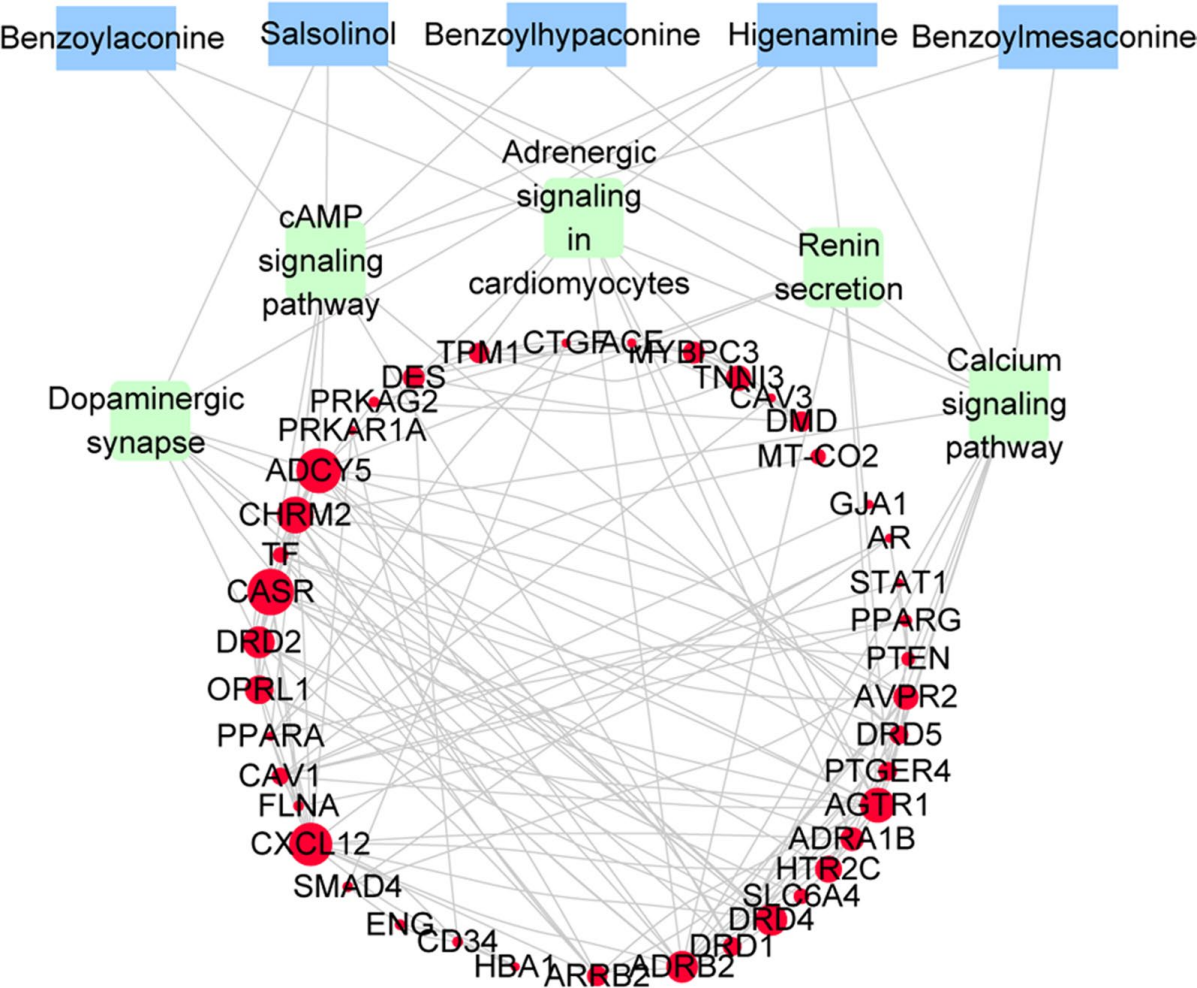
Protein–protein interaction network

124 predictive targets were obtained from BATMAN-TCM database. There were 212 related-targets of heart failure collected from HPO database. The interaction between drug targets and heart failure disease was constructed by String database and Cytoscape v3.5.1 (showed in Fig. 1). A total of 42 targets for anti-heart failure were found. The target was represented by a round node whose size represented degree (the number of interacting proteins in the network). In other words, the larger the node was, the more important it was to play a role in anti-heart failure networks. The top 10 targets for degree were CASR, ADCY5, CXCL12, CHRM2, AGTR1, DRD4, ADRB2, DRD2, OPRL1 and HTR2C. Among them, CHRM2 was the target of benzoylaconine, benzoylhypaconine, benzoylmesaconine and higenamine. DRD4, DRD2 and ADRB2 were the targets of higenamine and salsolinol.

#### Pathway enrichment analysis

There were eight main biological signal pathways obtained by pathway enrichment analysis of cardiac candidate targets. According to the p-value, it was cAMP signaling pathway (p = 1.41 × 10^−13^), Renin secretion (p = 1.02 × 10^−10^), Calcium signaling pathway (p = 8.87 × 10^−10^), Dopaminergic synapse (p = 5.80 × 10^−9^) and Adrenergic signaling in cardiomyocytes (p = 1.27 × 10^−8^), respectively. The smaller the p value was, the higher the correlation was.

Based on the analysis above, it was found that the pathways significantly influenced myocardial contraction and energy metabolism. In detail, cAMP signaling pathway, Dopaminergic synapse, Adrenergic signaling in cardiomyocytes, Calcium signaling pathway were closely related to myocardial contractility. cAMP signaling pathway and Calcium signaling pathway also affected mitochondrial energy metabolism. Therefore, the experimental study of NAP or HSP on heart function mainly focused on myocardial contractility and energy metabolism.

#### Cardiac effect analysis on an acute heart failure model

**Fig. 2:**
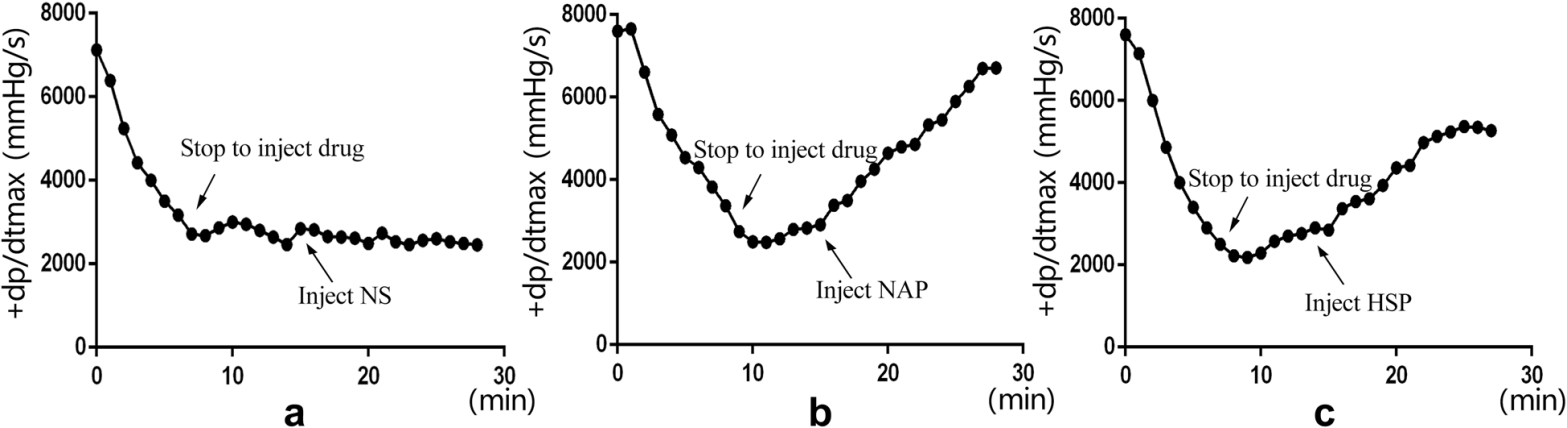
The results of NAP and HSP on acute heart failure (**a** NS group; **b** NAP group; **c** HSP group)

Figure 2 indicated that +dp/dtmax of rats was rapidly decreased after injecting the mixture of propafenone hydrochloride and nimodipine. Combined use of both could cause a rapid inhibition of cardiac function in a very short time. At 7–8 min, +dp/dtmax dropped more than 50% and the injection was stopped. The value did not rise significantly in 5 min, so the model was considered successful. After giving NS, it was clear that the value did not increase (Fig. 2a), which meant the damaged myocardial contractility did not recover. However, the value of +dp/dtmax climbed immediately when the extraction of NAP or HSP was given (Fig. 2b, c). The most amazing result was NAP almost restored the severely impaired myocardial contractility to normal level, while HSP only made it return to the level of 70–80%. These results fairly proved the superiority of NAP in an acute heart failure model.

#### Cardiac effect analysis on an Adriamycin heart failure model

**Fig. 3:**
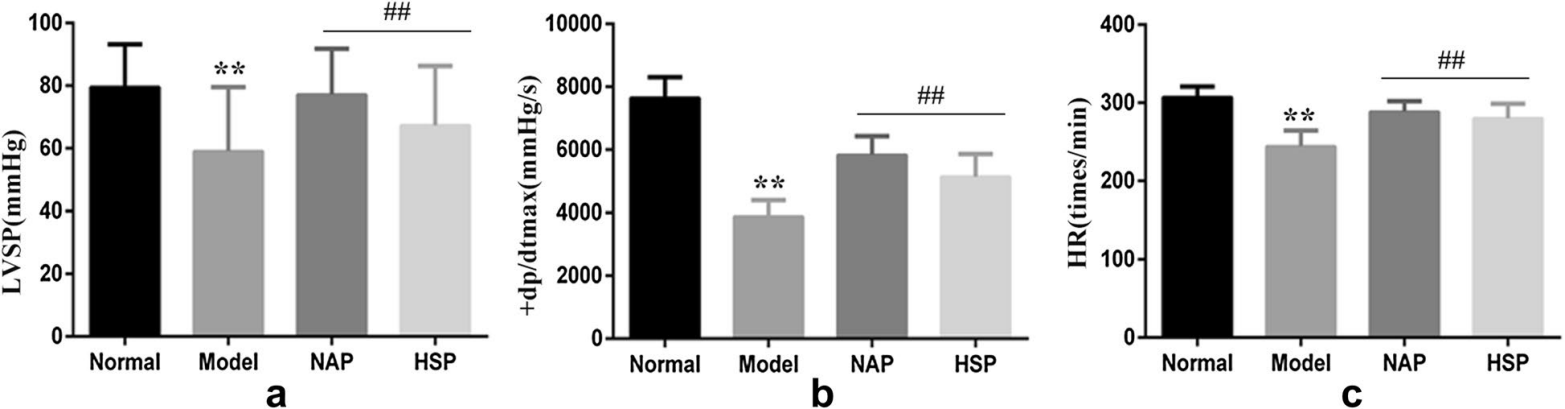
Effect of NAP and HSP on heart function of rats (**a** LVSP; **b** +dp/dtmax; **c** HR; vs normal group, **p < 0.01; vs model group, ^##^p < 0.01)

The effect of NAP and HSP on heart function indexes of rats was listed in Fig. 3. Comparing with normal group, LVSP, +dp/dtmax, and HR of model group rats were significantly dropped (p < 0.01) after continuous administration of Adriamycin, which indicated that cardiac function of rats was obviously inhibited. When given the extraction of NAP or HSP, LVSP, +dp/dtmax, and HR of rats increased greatly (p < 0.01), which meant the cardiac function improved dramatically. Overall, NAP showed a stronger cardiac function recovery than HSP.

#### Results of myocardial tissue ATPase and myocardial cell morphology

**Fig. 4:**
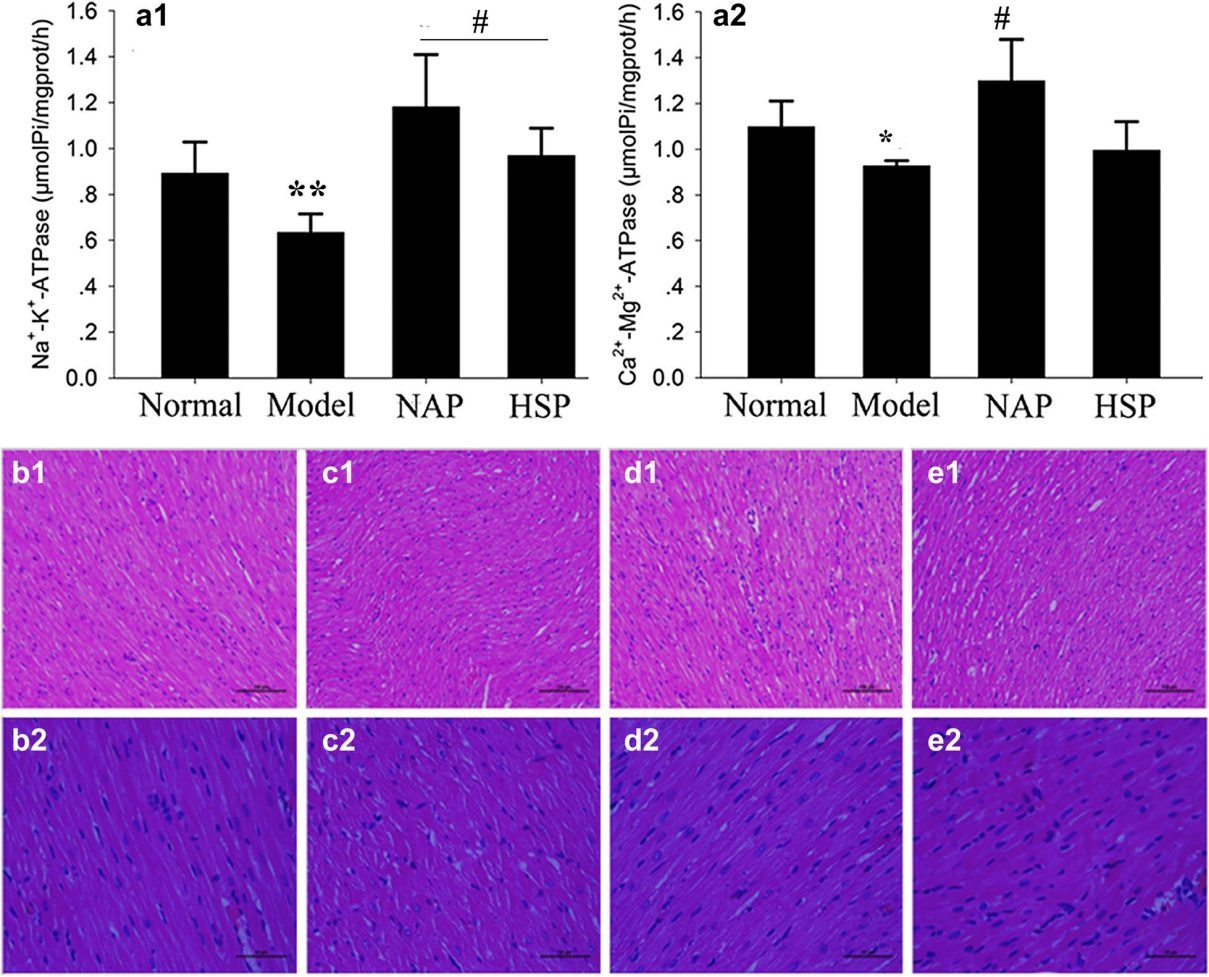
Results of *Na*^+^–*K*^+^–*ATPase* (**a1**) and *Ca*^*2*+^–*Mg*^*2*+^–*ATPase* (**a2**) in myocardial tissue and the pathological sections. Normal group (**b1** ×20, **b2** ×40), model group (**c1** ×20, **c2** ×40), NAP group (**d1** ×20, **d2** ×40), and HSP group (**e1** ×20, **e2** ×40), vs model group, *p < 0.05; vs normal group, ^#^p < 0.05, ^##^p < 0.01

The effect of NAP and HSP on ATPase content in rat myocardial tissues was shown in Fig. 4a. Comparing with normal group, the content of *Na*^+^–*k*^+^–*ATPase* and *Ca*^*2*+^–*Mg*^*2*+^–*ATPase* in model group went down significantly (p < 0.05). After giving NAP or HSP for 5 days, the content of *Na*^+^–*k*^+^–*ATPase* and *Ca*^*2*+^–*Mg*^*2*+^–*ATPase* went up sharply. Totally speaking, HSP group was near to the normal one, while NAP even exceeded the normal one.

Pathological analysis results were showed in Fig. 4b. It demonstrated that normal myocardial cells were arranged in order without deformation, necrotic cells or inflammatory cell infiltration. After Adriamycin administration, myocardial cells injured obviously with myocardial fiber disorder, thinning, and dissolution fracture. Some ones were even interstitial edema, accompanied by vacuole-like changed. After giving NAP or HSP, myocardial cells could be recovered to a certain extent. The arrangement tended to be close and vacuolar changes decreased. In particular, the morphology of NAP group approached to the normal group, which suggested the protective effect of NAP on myocardium was quite excellent.

#### Quantitative thermo-kinetic parameters for mitochondria growth

**Fig. 5:**
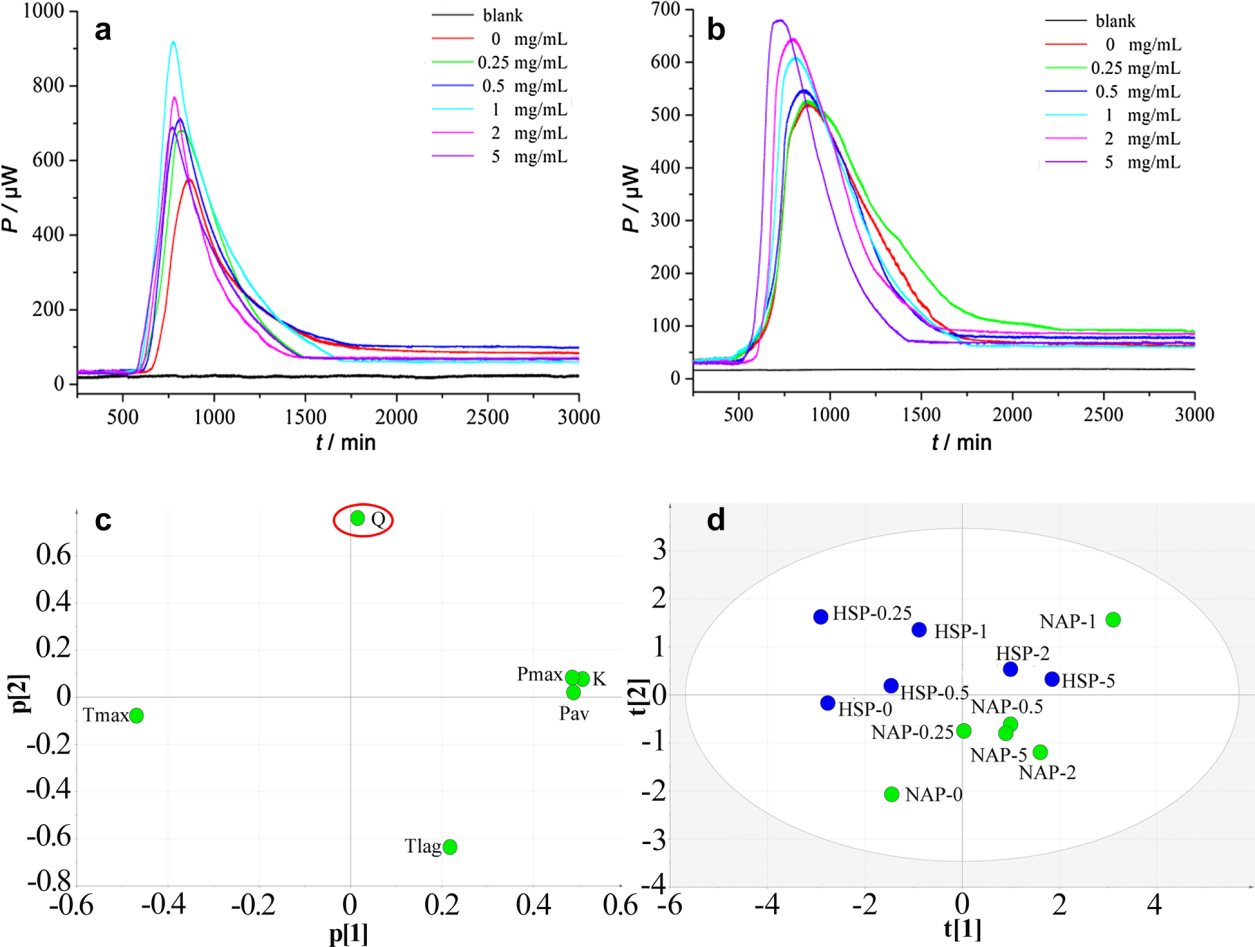
The results of HP-time curves of mitochondria growth at 37 °C and PCA analysis (**a** HP-time curves of mitochondria growth of NAP; **b** HP-time curves of mitochondria growth of HSP; **c** loadings plot shows the contribution of the original variables (thermometric parameters) for the first two principal components PC1 and PC2; **d** scores plot indicates the distribution of concentration of HSP and NAP, and blue and green scatter plots represent the different concentrations of HSP and NAP, respectively)

As shown in Fig. 5, the power-time curves of heat generation of mitochondria in the absence or presence of different concentrations of NAP or HSP were recorded. It could be found that the shape of curves in the administered groups (Ch 2–7) changed when compared with the control group (Ch 1). With the rising of concentration, the curve shape changed more significantly. However, the variation trends of NAP and HSP were not exactly the same. In detail, low concentrations (0–1 μg ml^−1^) in NAP group could promote the metabolism of mitochondria, while high concentrations (2–5 μg ml^−1^) posed an inhibitory effect. From Table 2, the thermodynamic parameters also made a similar performance. Most ones reached their peaks at a concentration of 1 μg ml^−1^, including k, P_max_, Q and P_av_. In HSP group, k, P_max_, Q, P_av_, T_lag_, and P_av_ of samples all increased continuously within 0–2 μg ml^−1^. When the applied concentration reached 5 μg ml^−1^, the value of some parameters began to decrease. These results clearly showed that the extraction of ALRP had a complex regulating action on mitochondria metabolism. Energy metabolism could be promoted by low concentrations and inhibited by high concentration. Interestingly, NAP could achieve a higher promotion effect at a lower concentration range.

**Table 2:** Quantitative thermo-kinetic parameters for mitochondria growth at 37 °C affected by NAP and HSP

Sample	c/μg ml^−1^	k/10^−3^ min^−1^	P_max_/μW	T_max_/min	Q/J	T_lag_/min	P_av_/μW
NAP	0	16.1	552.4	864.2	16.7	382.1	242.8
	0.25	17.6	680.7	824.3	17.2	294.1	299.3
	0.5	20.8	713.0	810.3	19.3	355.6	307.9
	1	28.5	919.8	775.5	22.8	291.9	331.6
	2	25.7	771.5	781.2	15.3	301.7	290.5
	5	21.1	690.6	771.3	16.6	294.2	293.1
HSP	0	14.2	522.7	884.2	17.2	211.5	229.5
	0.25	14.3	528.0	870.3	21.8	187.3	208.9
	0.5	15.4	547.8	847.2	19.0	244.8	274.7
	1	17.3	609.3	809.3	20.8	203.8	264.7
	2	23.0	645.9	799.0	20.1	274.5	326.3
	5	25.2	680.7	728.0	17.8	239.4	318.8

#### Principal component analysis (PCA) results

The effects of various concentrations of NAP and HSP on mitochondrial energy metabolism differed greatly. It was difficult to objectively determine the thermodynamic parameters represent the eigenvalues. Therefore, PCA was introduced to extract the main parameters which could represent the main change rule of data after dimensionality reduction.

SPSS 22.0 statistical software was carried out PCA analysis on six parameters. Loadings plot (Fig. 5c) indicated that Q was the most important thermodynamic index to distinguish the difference of mitochondrial metabolism between NAP and HSP, for it was the longest point from the origin. Scores plot (Fig. 5d) suggested that there was a good separation between NAP and HSP, though a few overlaps existed. These results also revealed the action diversity of two products. Through a visual analysis, total heat production of mitochondria peaked at 22.8 J when the concentration of NAP was 1 μg ml^−1^, and then began to decline dramatically. That of HSP also peaked at 20.8 J at the concentration of 1 μg ml^−1^, and then went down slowly. But the reduction speed was obviously lower. These results showed that the effect on promoting mitochondrial energy metabolism of NAP was better than HSP.

## Discussions

The mechanism of heart failure is rather complex and has not yet been fully elucidated. The core pathogenesis is considered as the abnormal of systolic function. The basic determinants of myocardial contraction include myocardial contractile protein, energy metabolism and excitation–contraction coupling [31]. Any change of these factors can lead to heart failure. Adriamycin can cause serious myocardial toxicity, and the damage degree is highly correlated with dose [32]. The main poisoning mechanisms are oxidative stress and mitochondrial dysfunction [33]. It can induce lipid peroxidation injury, and produce malondialdehyde and other metabolites. These substances damage the integrity of the myocardial cell membrane and the mitochondrial membrane, and finally result in cell autolysis and the destruction of contractile protein [34]. Meanwhile, Adriamycin can inhibit the activity of *Na*^+^–*K*^+^–*ATPase* in cell membranes and weaken the activity of *Ca*^*2*+^–*ATPase* in sarcoplasmic reticulum membranes. The direct result is that myosin does not decompose ATP normally [35]. Propafenone hydrochloride is a classic sodium channel blocker [36], while nimodipine blocks the calcium channels [37]. Their combination can not only interfere with the sodium influx during depolarization, but also block the calcium influx during the plateau, and contribute to the myocardial excitation–contraction coupling disorder. So these two models greatly reflect the basic pathogenesis of heart failure.

Multiple pathways found in network pharmacology analysis play an important role in the treatment of heart failure. The most impressive one is cAMP signaling pathway. When it works, adenylate cyclase is activated, and ATP is converted to cAMP [38]. It makes the receptor dependent calcium channel open and promote the increase of intracellular Ca^2+^ concentration [39]. This is an important mechanism to enhance myocardial contractility. It can also strengthen the beta oxidation of fatty acids and improve the energy metabolism of hypertrophic cardiomyocytes [40, 41]. In addition, this pathway can further pose an effect on the Renin pathway [42]. Another pathway to be mentioned is adrenergic signaling in cardiomyocytes. It also inspires adenylate cyclase and promotes ATP catabolism [43]. Calcium signaling pathway is a downstream one, which enables to boost the intracellular calcium concentration and act on the mitochondrial calcium uniporter [44]. When this network is activated by ALRP, myocardial contractility and energy metabolism should be improved comprehensively.

Higenamine and salsoline play a crucial role in the myocardial contractility. Higenamine is a full agonist of β-1 adrenergic receptor [45]. Meanwhile, it is confirmed to have β-2 adrenergic receptor agonist activity [46]. Salsolinol is a partial agonist for β-receptor [45]. According to our results, these two components in NAP were about 5–32 times as much as HSP (Additional file 2). The results of two animal experiments also proved the beneficial effects of the increase in components’ contents on myocardial contractility. Monoester diterpenoid alkaloids are the key to energy metabolism of mitochondria [10, 47]. Previous study found that the abundance in NAP was 2–14 times over that of HSP, an Additional file shows this in more detail (see Additional file 2). Likewise, NAP performed a better effect on energy metabolism and produced more thermal energy in a lower concentration. Moreover, our tests also demonstrated the antagonistic effect of ALRP on Adriamycin induced myocardial injury. Although the effects and mechanisms are not yet clear, the increase in total alkaloids is benefit to enhance the therapeutic effect.

## Conclusions

This study demonstrates the advantages of NAP with high alkaloid contents in treating heart failure, which provides sufficient scientific evidence for its industrial development.

## Additional files

**Additional file 1.** Minimum Standards of Reporting Checklist.

**Additional file 2.** The Content Determination of Eight Alkaloids in NAP and HSP by HPLC-MS/MS.

## Abbreviations

HF: heart failure
PHI: propafenone hydrochloride injection
NS: normal saline
NI: nimodipine injection
HR: heart rate
ALRP: Aconiti Lateralis Radix Praeparata
HSP: heishunpian
DDAs: diester diterpenoid alkaloids
MDAs: monoester diterpeniod alkaloids
LVSP: left ventricular systolic pressure
+dp/dtmax: left ventricular maximum pressure rising rate
NAP: a novel processed product of *Aconiti Lateralis Radix Praeparata*
