# Supplementary material for: Cardiac function evaluation for a novel one-step detoxification product of Aconiti Lateralis Radix Praeparata
Source: Chin Med. 2018 Dec 17;13:62. doi: 10.1186/s13020-018-0219-4 (PMC6297959; doi:10.1186/s13020-018-0219-4)
Supplement: Supplementary file 2 — Additional file 2. The Content Determination of Eight Alkaloids in NAP and HSP by HPLC-MS/MS. [file 13020_2018_219_MOESM2_ESM.docx]

**Additional files 2**

**the Content Determination of Eight Alkaloids in NAP and HSP by HPLC-MS/MS**

**HPLC-MS/MS Analysis**

**Materials**

Standards of benzoylmesaconine, benzoylaconine, benzoylhypaconine, mesaconitine, aconitine and hypaconitine were purchased from the National Institute for the Control of Pharmaceutical and Biological Products of China, Beijing, China, and the lot numbers were 110799-201106, 110720-201111, 110798-201106, 111795-200901, 111794-200901, and 111796-201303, respectively. Standards of higenamine and salsolinol were supplied by Chengdu Pufei De Biotech Co., Ltd. (Chengdu, China), and the lot numbers were 141111and 140922, respectively. The purity of all the standards was greater than 98.0%.

**MS Conditions**

Break HSP and NAP to powder through the three-sieve. A total of 0.2 g of each powder was accurately weighed and extracted with 10 mL of methanol solution by ultrasonic extraction (40Hz, 300W) for 30 min. The extracted solution was cooled, which contributed to weight loss during the extraction procedure, and filtered through a 0.22 μm micropore film to yield the subsequent filtrate solution. Then we obtained the sample solution for HPLC-MS/MS analysis.

Samples were analyzed by an Agilent1260 high performance liquid chromatograph and Agilent6460C triple-quadrupole tandem mass spectrometry (Agilent Technologies, Santa Clara, CA, USA) using a Phenomenonex Gemini C18 column (4.6 mm×150 mm, 5 μm). The column temperature was 30 °C and 0.5 μL of the sample solution was injected into the system. The mobile phase was composed of (A) 0.1 % aqueous formic acid in water and (B) acetonitrile using a gradient program of 2 % B for 0 – 1 min, 20 – 50 % B for 1 – 5 min, 50 – 70 % B for 5 – 10 min,75 – 100 % B for 10 – 15 min, with a mobile flow rate of 0.45 ml/min.

Mass spectrometric scan were obtained by electrospray ionization (ESI) in positive-ion mode with a scanning interval 100-1000 m/z. The main parameters for MS were set as follows: gas temperature, 300 ° C; gas flow, 11 L/min; nebulizer, 35 psi; capillary voltage, 4000 V; atomizer pressure 15 psi (1 psi = 6.895 Kpa). MS parameters and MRM transitions of each analyte are shown in table 1. This method had been verified in another article and we deemed it met our needs[[17](#_ENREF_17)].

**Table S1 Detected ion pairs of fourteen alkaloids**

| Name | m/z | Fragmentor (V) | Collision energy (ev) |
| --- | --- | --- | --- |
| aconitine | 646.3/105.1 | 180 | 50 |
| mesaconitine | 632.3/105.1 | 180 | 46 |
| hypaconitine | 616.3/105.0 | 180 | 46 |
| benzoylaconine | 604.3/105.0 | 180 | 45 |
| benzoylmesaconine | 590.3/105.0 | 205 | 46 |
| benzoylhypaconine | 574.3/105.0 | 200 | 46 |
| higenamine | 272.3/106.9 | 110 | 16 |
| salsolinol | 180.2/117.0 | 100 | 20 |

**Preparation of Standard solution**

The mixed standard containing The mixed standard containing 1.509 ug/ml aconitine , 1.102 ug/ml mesaconitine, 1.022 ug/ml hypaconitine, 1.362 ug/ml benzoylaconine, 1.343 ug/ml benzoylmesaconine, 1.218 ug/ml benzoylhypaconine, 1.134 ug/ml higenamine and 1.216 ug/ml salsolinol was prepared stock into a volumetric flask and dissolved with 10 mL methanol. These solutions were stored in dark glass bottles at 4 °C and stable for at least 1 week. Working standard solutions were freshly prepared by diluting suitable amounts of the above solutions with methanol before injection.

**Results**

In this two processed-products, the content of toxic components (aconitine，mesaconitine，hypaconitine ) was almost similar. The content of other alkaloids in NAP was higher than that of HSP, especially benzoylhypaconine, higenamine and salsolinol. The results showed in table 2. These results illustrated that this novel method could achieve the same detoxification effect as traditional methods. Additionally, it also avoided a large loss of alkaloids and retained active ingredients as much as possible.

**Table S2 determined results of 11 investigated compounds of HSP and NAP（，n=6）**

| **Alkaloids** | **Regression equations** | **R^2^** | **linear ranges (µg/mL)** | **LOD (ng/mL)** | **LOQ (ng/mL)** | **HSP**  **(µg/mL)** | **NAP**  **(µg/mL)** |
| --- | --- | --- | --- | --- | --- | --- | --- |
| aconitine | Y=58581X+868.92 | 0.9990 | 0.0204 - 0.6520 | 0.79 | 3.20 | 1.8 | 2.18 |
| mesaconitine | Y=105390X+250.14 | 0.9999 | 0.0045- 0.1440 | 0.77 | 3.02 | 30.17 | 31.16 |
| hypaconitine | Y=63010X+575.21 | 0.9989 | 0.0071 - 0.2260 | 0.70 | 2.70 | 40.65 | 61.74 |
| benzoylaconine | Y=55701X+21876 | 0.9954 | 0.1750 - 5.6000 | 0.71 | 2.91 | 7.04 | 53.38 |
| benzoylmesaconine | Y=122753X+5122.4 | 0.9975 | 0.0316 - 1.0100 | 0.74 | 3.09 | 208.89 | 648.38 |
| benzoylhypaconine | Y=80577X+11748 | 0.9963 | 0.0384 - 1.2300 | 0.87 | 3.37 | 23.26 | 366.99 |
| higenamine | Y=5356X+155.42 | 0.9927 | 0.0084 - 0.1340 | 1.79 | 7.32 | 0.07 | 2.27 |
| salsolinol | Y=9928.2X-51.282 | 0.9983 | 0.0065 - 0.4160 | 1.62 | 6.56 | 72.42 | 386.66 |

Note: LOQ: Limit of Quantity; LOD: limit of detection.
